# Multidimensional assessment of diaphragmatic dysfunction in late-onset Pompe disease: a prospective cohort study

**DOI:** 10.1186/s13023-026-04343-0

**Published:** 2026-04-07

**Authors:** Ana Hernández-Voth, Javier Sayas-Catalán, Marta Corral-Blanco, Miguel Jiménez-Gómez, Gema Carvajal-Cuesta, Cristina Domínguez-González, Laura Bermejo-Guerrero, Paloma Martín-Jiménez, Victoria Villena-Garrido

**Affiliations:** 1 Mechanical Ventilation Unit, Pulmonology Department, 12 de Octubre University Hospital, Avenida de Córdoba, no number, Madrid, 28041 Spain; 2 Respiratory Diseases Research Group, 12 de Octubre Hospital Research Institute (I+12), Madrid, Spain; 3 Department of Medicine, Universidad Complutense de Madrid, Madrid, Spain; 4 Neurology Department, 12 de Octubre University Hospital, Madrid, Spain

**Keywords:** Late-onset Pompe disease, Diaphragmatic ultrasound, Respiratory function, Enzyme replacement therapy

## Abstract

Late-onset Pompe disease (LOPD) often leads to progressive respiratory failure due to diaphragmatic involvement. Conventional pulmonary function tests frequently fail to detect early respiratory muscle weakness. We conducted a five-year prospective cohort study of 15 genetically confirmed LOPD patients to evaluate diaphragmatic function using a multidimensional approach that included spirometry, inspiratory/expiratory pressures (MIP/MEP), sniff nasal inspiratory pressure (SNIP), arterial blood gases, and diaphragmatic ultrasound. Data were analyzed using generalized linear mixed models. Forced vital capacity (FVC) declined progressively (− 1.05% per visit, *p* = 0.010), while diaphragmatic thickening fraction (TF, − 3.46% per visit, *p* < 0.0001), SNIP (− 2.53 cmH₂O per visit, *p* = 0.003), and MIP (− 3.56% per visit, *p* < 0.001) showed earlier and steeper declines. TF and SNIP were significantly more sensitive than FVC in detecting early respiratory involvement (*p* < 0.05). Patients receiving enzyme replacement therapy (ERT) had slower functional decline, with significantly higher SNIP values (*p* = 0.003). One patient developed symptomatic diaphragmatic weakness despite preserved FVC. SNIP and TF are more sensitive than FVC for early detection of respiratory dysfunction in LOPD and may allow earlier initiation of enzyme replacement therapy and respiratory support.

**Supplementary Information:**

The online version contains supplementary material available at 10.1186/s13023-026-04343-0.

## Introduction

Pompe disease (Glycogen Storage Disease II, OMIM #232300) is an autosomal recessive disorder caused by mutations in the gene encoding acid alpha-glucosidase. Historically, the prevalence was estimated to be approximately 1 in 40,000 births. However, its estimated prevalence has increased in recent years with the implementation of newborn screening programs, suggesting that the frequency may be substantially higher, ranging from 1:4,447 to 1:37,094 depending on the population studied, ethnic background, and screening methodology [1].

Late-onset Pompe disease (LOPD) is defined by symptom onset after the first year of life and preservation of cardiac function, and it primarily affects skeletal and respiratory muscles. Because LOPD often presents with mild and nonspecific symptoms, diagnostic delay is common. Previous epidemiological studies have reported a mean delay of approximately six years between symptom onset and definitive diagnosis. The increasing use of newborn screening programs and the development of more sensitive diagnostic algorithms have contributed to reducing this delay and allow identification of patients even in presymptomatic stages [2].

In clinical practice, patients are usually classified as symptomatic or asymptomatic depending on the presence of skeletal muscle weakness, respiratory symptoms, or objective evidence of respiratory muscle involvement. Current therapeutic strategies generally recommend initiating enzyme replacement therapy (ERT) in symptomatic patients, particularly when there is evidence of respiratory muscle dysfunction or progressive muscle weakness. The clinical course is heterogeneous; in some patients, respiratory involvement may precede limb-girdle muscle weakness, potentially leading to chronic respiratory failure [3].

Early identification of respiratory involvement is therefore particularly important, as it may influence clinical monitoring strategies and the timing of therapeutic interventions. Respiratory dysfunction in LOPD is multifactorial. Neurological impairment of the respiratory muscles is well documented. Additionally, emerging evidence supports the presence of both obstructive sleep apnea and central hypoventilation, likely related to glycogen accumulation in central respiratory centers within the brainstem [4–8]. Although skeletal muscles are broadly affected, the diaphragm appears to be particularly vulnerable. This has been demonstrated in studies assessing transdiaphragmatic pressure through invasive manometry during phrenic nerve magnetic stimulation [9], as well as in histological findings from animal models showing extensive glycogen storage in diaphragmatic fibers [10, 11]. These data underscore the central role of diaphragmatic dysfunction in the physiopathology of LOPD.

Enzyme replacement therapy (ERT) has shown benefits in stabilizing or mildly improving pulmonary function. Two ERT formulations are currently available for LOPD: alglucosidase alfa (Myozyme^®^, Sanofi Genzyme) and the more recently developed avalglucosidase alfa (Nexviazyme^®^, Sanofi Genzyme), approved by the United States Food and Drug Administration (FDA) in August 2021 and by the European Medicine Agency in June 2022 due to its enhanced cellular uptake. More recently, the combination of cipaglucosidase alfa plus miglustat (Pombiliti^®^ plus Opfolda^®^, Amicus Therapeutics) was approved by the FDA in 2023 for the treatment of adults with LOPD weighing ≥ 40 kg who are not improving on their current ERT. However, once respiratory failure is established, non-invasive mechanical ventilation (NIV) remains the only supportive intervention [12–16]. Respiratory physiotherapy also represents an important component of the multidisciplinary management of LOPD, contributing to airway clearance, respiratory muscle conditioning, and prevention of respiratory complications. Conventional pulmonary function tests, including forced vital capacity (FVC), often lack the sensitivity to detect early diaphragmatic weakness. A systematic review showed that over 30% of the LOPD patients requiring NIV had a normal FVC, highlighting the risk of underdiagnosing early respiratory compromise when relying solely on spirometry [17].

Because respiratory failure is the leading cause of mortality in LOPD, early detection of diaphragmatic weakness is critical [18]. Therefore, the aim of the study was to longitudinally compare multiple respiratory parameters, including standard pulmonary function tests and diaphragmatic ultrasound measurements, in order to characterize respiratory disease progression in patients with LOPD.

## Materials and methods

This was a prospective, observational cohort study conducted at the Neuromuscular Disease Unit of Hospital Universitario 12 de Octubre (Madrid, Spain). The study period ranged from 2018 to 2024, with follow-up assessment scheduled every six months.

Eligibility criteria included genetically confirmed LOPD, defined by the presence of pathogenic variants in the GAA gene and reduced acid alpha-glucosidase enzymatic activity according to standard diagnostic criteria; age ≥ 18 years; and the ability to perform respiratory function tests and diaphragmatic ultrasound assessments.Exclusion criteria included refusal or inability to provide informed consent.

Due to the rarity of the disease, all eligible patients followed at the center during the study period were included. No a priori sample size calculation was performed. All patients underwent a baseline assessment and then every six months for up to five consecutive years. Each visit included the following assessments.

- Clinical data: body mass index (BMI), ERT status (and time since the beginning if treated), presence of orthopnea and/or symptoms suggestive of nocturnal hypoventilation (morning headache, daytime somnolence and fatigue).
- Pulmonary function tests: upright and supine FVC (% of predicted value) (JaegerMasterScope^®^, CareFusion, Hoechberg, Germany); maximum inspiratory pressure (MIP) (% of predicted value); maximum expiratory pressure (MEP) (% of predicted value) and sniff nasal inspiratory pressure (SNIP) (cmH_2_O) (MicroRPM^®^, CareFusion, Hoechberg, Germany), arterial blood gases (mmHg) (Gem Premier 4000 ^®^, Instrumentation Laboratory, Bedford, MA, USA).
- Diaphragmatic ultrasound (Ultrasound Digital Color Doppler X3, Sonoscape Medical Corp, Guangdong, China) was performed in a 90-degree position using a high-frequency linear probe. Diaphragmatic thickness was measured at vital capacity and residual functional capacity, and thickening fraction (TF) was calculated as the relative increase in thickness during inspiration [19].
- NIV parameters and indicators: NIV daily use (hours) was registered using ResScan^®^ (ResMed, Australia), VIVO 50^®^ (Breas Medical, Sweden), and Care Orchestrator^®^ (Philips, Murrysville, PA, USA).

To minimize measurement bias, the same trained physicians and technicians conducted all assessments using the same protocols and equipment.

ERT was administered in symptomatic patients with LOPD, in accordance with the approved indication stated in the summary of product characteristics. It consisted of the intravenous administration of alglucosidase alfa at a dose of 20 mg/kg every two weeks, in accordance with the prescribing information approved by regulatory agencies [20]. Continuation of therapy was guided by clinical assessment of efficacy and tolerability throughout the study period. For patients who showed clinical progression despite treatment with alglucosidase alfa, therapy was switched to avalglucosidase alfa following its regulatory approval.

### Ethics

The study protocol was approved by the Research Ethics Board under the number CEIm 19/100 and complied with the Declaration of Helsinki. All participants provided written informed consent prior to enrollment.

### Statistical analysis

Descriptive statistics were expressed as frequencies, means, and standard deviations. To assess the progression of respiratory function over time, generalized linear mix models (GLMMix) were applied, adjusting for baseline values. Subsequently, the evolution of each variable was further analyzed in relation to sex, age at diagnosis (< 40 vs. ≥40 years), and use of NIV, by generating separate regression models for each subgroup. In addition, to evaluate the impact of ERT, patients were categorized into two groups: those with stable ERT initiated before study inclusion (“treated”) and those who started ERT during follow-up or remained untreated (“untreated”).

All statistical analysis were performed using the R statistic software (R Core Team, 2024) with the interface R Studio. In addition to the standard functions of Rbase ^®^ for the processing data, the following data packages were used: tidyr ^®^ and dplyr ^*®*^ for the data management, lme4 ^®^ and lmerTest ^®^ for the mixt models, and ggplot2^®^ for the graphic design.

Missing data were handled using complete-case analysis, and no imputation was performed.

## Results

A total of 15 patients with genetically confirmed LOPD were included. The mean age at symptom onset was 33.3 years-old (SD 15.2, range 26–47), age at diagnosis was 40.4 years-old (SD 11.9, range 36–49), and ten participants were male (67%). General demographic and clinical characteristics are described in Table 1.

**Table 1:** General characteristics of the studied patients

Variable		(*n* = 15)
Male, n (%)		10 (67%)
Age at diagnosis (years), median (IQR)		49 (43.5–58)
Mechanical ventilation, n (%)	Yes, before the study Yes, during the study No	6 (40%) 1 (7%) 8 (53%)
ERT treatment, n (%)	Yes, before the study Yes, during the study	10 (67%) 2 (13%)
	No	3 (20%)
ERT treatment (years), median (IQR)		7.38 (0.67–9.75)

ERT: Enzymatic replacement treatment; IQR: interquartile range

Seven patients (47%) completed the full 5-year follow-up, while others were followed for shorter periods: one for 4 years, two for 3 years, and four for 1 year.

At baseline, six patients (40%) were already on NIV. During the follow-up period, one previously asymptomatic, untreated patient developed orthopnea, despite preserved FVC. Subsequent nocturnal transcutaneous capnography revealed nocturnal hypoventilation, indicating a respiratory-onset presentation of LOPD.

### Respiratory function trends

Longitudinal changes in respiratory function are summarized in Table 2, with individual patient trajectories shown in Supplementary material 1. Key findings are detailed below.

**Table 2:** Evolution of the respiratory evaluations during the follow-up of the studied patients

Visits	1 (*n* = 15)	2 (*n* = 15)	3 (*n* = 11)	4 (*n* = 11)	5 (*n* = 10)	6 (*n* = 10)	7 (*n* = 7)	8 (*n* = 7)	9 (*n* = 7)	10 (*n* = 7)
FVC (%) Mean ± SD	84 ± 26	79 ± 24	86 ± 22	86 ± 27	82 ± 24	82 ± 23	78 ± 26	80 ± 23	74 ± 22	77± 25
FVC supine (%) median (range)	-6 (1–60)	-10 (1–60)	-6 (2–60)	-12 (1–60)	-14 (4–60)	-14 (0–60)	-13 (6–19)	-13 (10–23)	-17 (9–17)	-17 (9–19)
MIP (%) Mean ± SD	57 ± 21	56 ± 18	53 ± 21	51 ± 22	47 ± 21	44 ± 23	37 ± 21	30 ± 18	29± 16	31 ± 19
MEP (%) Mean ± SD	62 ± 20	54 ± 19	47 ± 16	51 ± 19	44 ± 18	46 ± 19	41 ± 20	33 ± 9	33 ± 8	40 ± 8
SNIP (cmH_2_O) Mean ± SD	65 ± 27	57 ± 17	60 ± 18	43 ± 17	43 ± 13	42 ± 15	32 ± 12	29 ± 11	31 ± 13	35 ± 13
pCO_2_ (mmHg) mean ± SD	40 ± 5	38 ± 6	37 ± 6	39 ± 6	35 ± 6	38 ± 5	38 ± 7	38 ± 6	40 ± 7	40 ± 6
TF (%) mean ± SD	65 ± 35	49 ± 21	54 ± 28	45 ± 19	43 ± 19	43 ± 22	32 ± 18	32 ± 16	32 ± 16	31± 15
NIV use (hours), mean ± SD	6 ± 2	7 ± 2	6 ± 2	6 ± 2	6 ± 2	6 ± 4	6 ± 3	7 ± 2	7 ± 3	7 ± 3

FVC: forced vital capacity, SD: standard deviation, MIP: maximum inspiratory pressure, MEP: maximum expiratory pressure, SNIP: sniff nasal inspiratory pressure, PF: peak flow, tcpCO2: transcutaneous pCO2, TF: thickening fraction, US: ultrasound, NIV: non-invasive ventilation

**Fig. 1:**
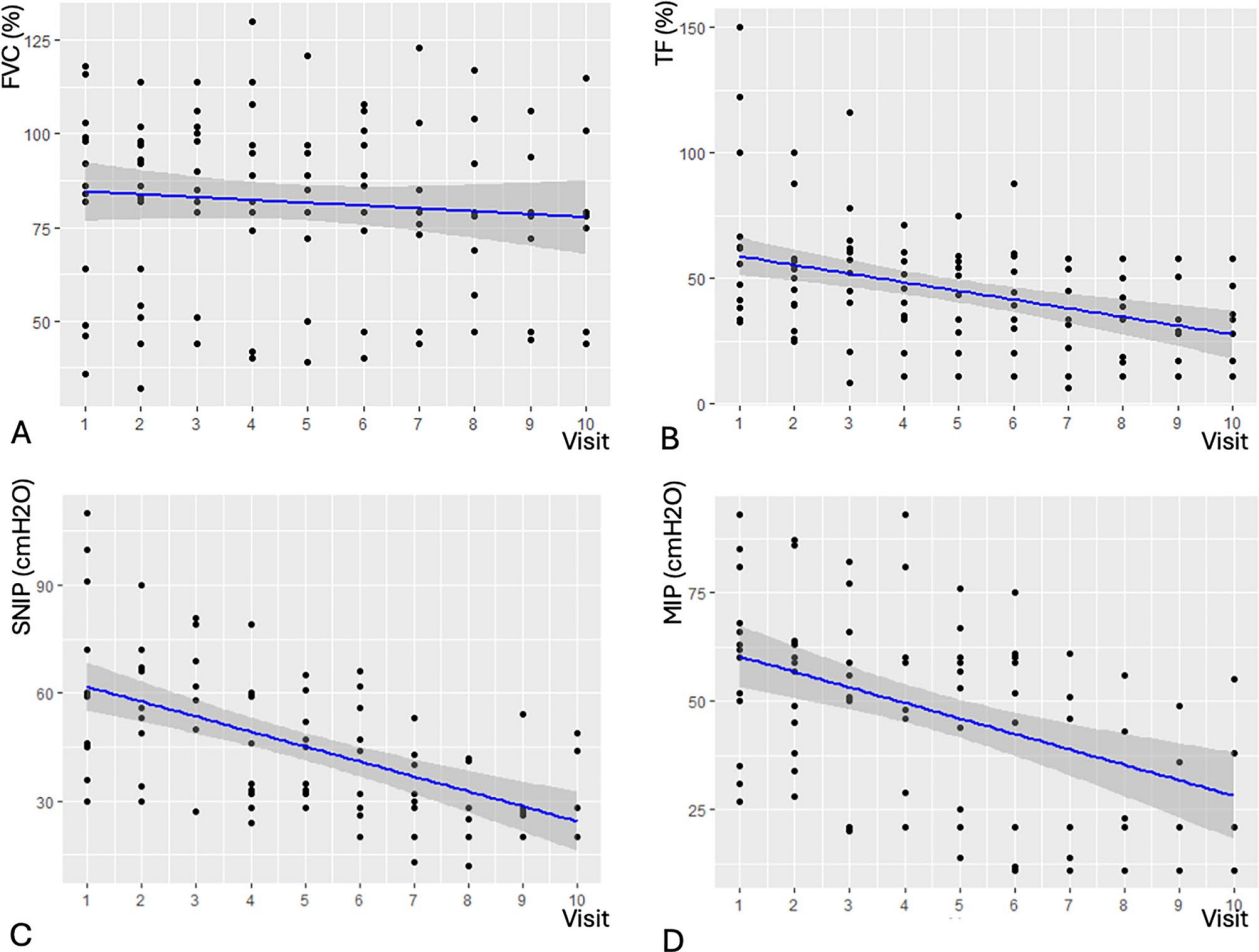
Longitudinal evolution of respiratory parameters reflecting diaphragmatic function in the studied population across follow-up visits: forced vital capacity (FVC, **1 A**), diaphragmatic thickening fraction (TF, **1B**), sniff nasal inspiratory pressure (SNIP, **1 C**) and maximum inspiratory pressure (MIP, **1D**)

FVC declined progressively over time (Fig. 1A), with a mean reduction of 1.05% per visit (*p* = 0.010). Using a GLMMix adjusted for baseline values, FVC trajectories remained significantly negative (0.87%, *p* < 0.0001) with no significant differences according to sex (*p* = 0.28), age at diagnosis (*p* = 0.50), or NIV status (*p* = 0.10).

Diaphragmatic TF declined more rapidly than FVC (Fig. 1B), with a mean decrease of 3.46% per visit (*p* < 0.0001). According to the adjusted GLMMix, each percentage point of baseline TF translated into only 0.24% at subsequent visits (*p* < 0.0001). Subgroup analyses using regression models showed no statistically significant differences in TF decline when stratified by sex (*p* = 0.64), age at diagnosis (< 40 vs. ≥40 years; *p* = 0.91), or use of NIV (*p* = 0.23).

Both SNIP and MIP showed steady declines throughout follow-up (Figs. 1C–D). SNIP decreased by 2.53 cmH₂O per visit (*p* = 0.003), while MIP declined by 3.56% per visit (*p* < 0.001). These trajectories remained significant after baseline adjustment and were not modified by sex, age at diagnosis, or NIV requirement.

#### Respiratory variables according with ERT treatment

To assess the potential impact of ERT on respiratory function, patients were categorized into two groups: those receiving stable, uninterrupted ERT prior to study inclusion (“treated”, *n* = 10, 67%) and those who initiated ERT during follow-up of remained untreated throughout the study (“untreated”, *n* = 5).

At baseline, the median duration of ERT with alglucosydase alfa among treated patients was 7.38 years (range 0.67–9.75). By the end of the study, seven patients had switched therapy: sit to avalglucosidase alfa and one to cipaglucosidase plus miglustat.

**Fig. 2:**
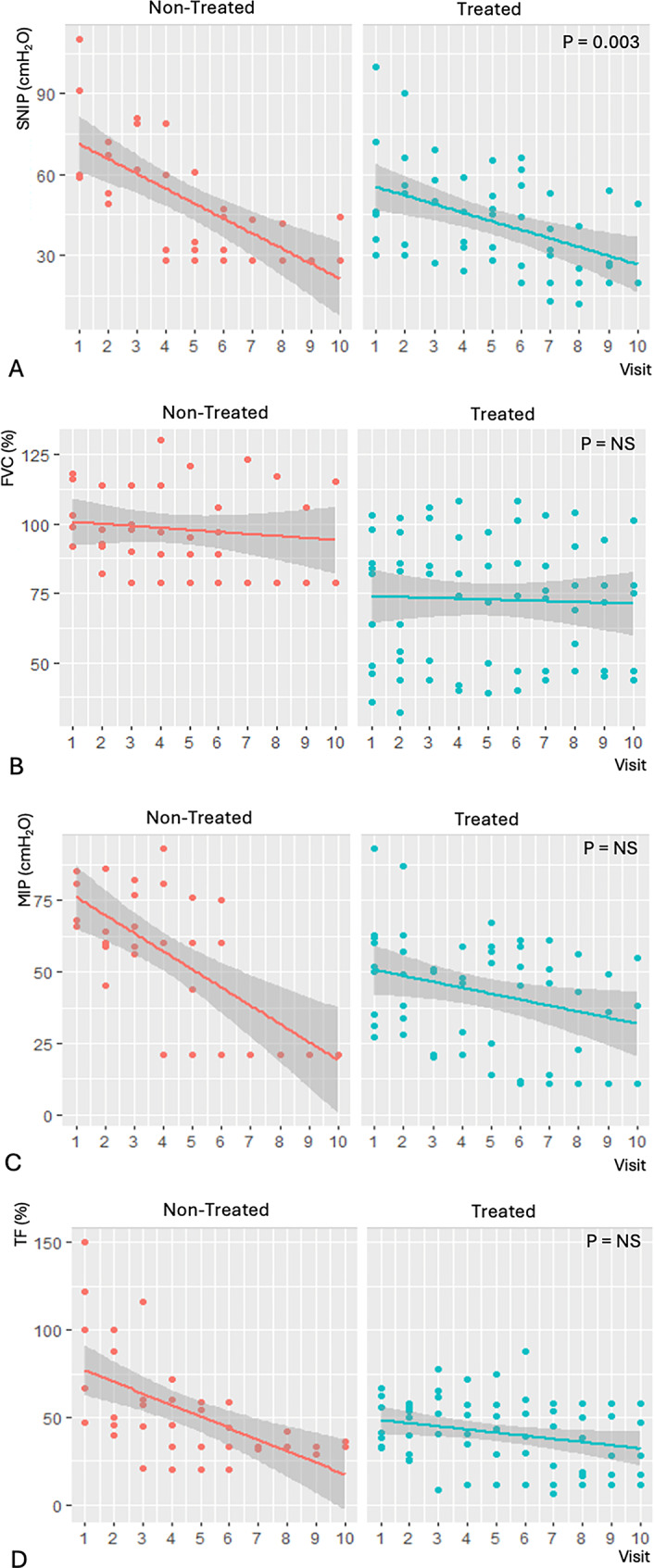
Comparison of respiratory parameters related to diaphragmatic function according to enzyme replacement therapy status across follow-up visits: (**A**) sniff nasal inspiratory pressure (SNIP), (**B**) forced vital capacity (FVC), (**C**) maximum inspiratory pressure (MIP), and (**D**) diaphragmatic thickening fraction (TF)

At the end of the follow-up, the adjusted GLMMix model showed that treated patients, compared to untreated patients, had higher mean values of FVC (+ 5.43%, *p* = 0.43), TF (+ 12.5%, *p* = 0.30), and MIP (+ 1.70%, *p* = 0.79), though these differences were not statistically significant (Fig. 2). In contrast, SNIP values were significantly higher in treated patients, with a mean difference of + 15.41 cmH_2_O (*p* = 0.003) (Fig. 2).

Among untreated patients, respiratory function declined more markedly. Notably, reductions in TF (from 78 to 32%), SNIP (from 86 to 28 cmH_2_O), and MIP (73 to 21 cmH_2_O) preceded the decline in conventional FVC (from 98 to 79%).

Importantly, the difference between FVC and TF became statistically significant as early as the first year of follow-up (*p* = 0.049), supporting the greater sensitivity of diaphragmatic ultrasound in detecting early respiratory compromise.

### Evolution of the studied respiratory variables during follow-up

Throughout the follow-up period, in all patients (both treated and untreated) diaphragmatic ultrasound TF (from 65% to 32%), SNIP (from 65 to 35 cmH₂O), and MIP (from 57% to 31%) demonstrated a more pronounced and earlier decline than FVC (from 84% to 77%), indicating their superior sensitivity in detecting diaphragmatic dysfunction (Table 2). Statistically significant differences between FVC and both SNIP (*p* = 0.032) and TF (*p* = 0.016) emerged as early as the first year of follow-up, while the divergence between FVC and MIP reached statistical significance in the second year (*p* = 0.035).

**Fig. 3:**
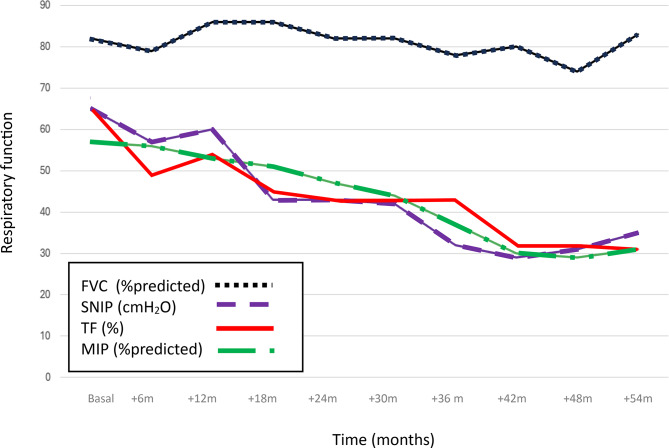
Longitudinal evolution (in years) of median respiratory parameters reflecting diaphragmatic function in the study population: maximum inspiratory pressure (MIP), forced vital capacity (FVC), diaphragmatic thickening fraction (TF), and sniff nasal inspiratory pressure (SNIP)

Overall, both inspiratory pressure-based parameters (SNIP and MIP) and TF exhibited a consistent and steeper decline over time, preceding changes in traditional spirometry measurements (FVC) (Fig. 3).

## Discussion

In this prospective cohort, we observed a consistent and progressive deterioration of respiratory function in patients with LOPD. While FVC declined slowly, SNIP, MIP and particularly TF exhibited earlier and steeper reductions, supporting their superior sensitivity in detecting early respiratory compromise.

Our findings are in line with priori studies, reporting FVC impairment in approximately 60% of LOPD patients, with 30–40% showing at least moderate reductions [21, 22]. In our cohort, 60% of patients had reduced FVC and 40% required NIV at baseline, reinforcing the significance of respiratory involvement even in the absence of profound muscle weakness [23].

Previous literature has described annual declines of 3% in maximal MIP and MEP among LOPD patients [24]. Our cohort suggest a more pronounced progression(-5.5% per year in MIP and 5.6% in MEP-). This may reflect more advanced disease stages at enrolment or the higher sensitivity of our multidimensional monitoring protocol.

Among our most notable findings, as in earlier descriptions, SNIP has demonstrated to be a noninvasive useful test detecting early neuromuscular diaphragmatic weakness, it can be performed by patients with advanced disease, and it gives prognostic information [25]. While SNIP has extensively validated in amyotrophic lateral sclerosis [26] and other neuromuscular diseases [27], this is, to our knowledge, the first study to report its longitudinal progression in LOPD. We recorded a mean SNIP reduction of − 7.2 cmH_2_O/year, confirming its value in detecting subclinical respiratory deterioration even when FVC remains within normal limits.

Beyond its diagnostic value, SNIP was the only respiratory parameter that showed a statistically significant difference between treated and untreated patients, with higher values in those receiving stable ERT. This suggests a potential treatment effect specifically detectable through SNIP, which may reflect its higher sensitivity to subtle improvements in diaphragmatic function. While FVC, TF, and MIP also trended better in the treated group, these differences did not reach statistical significance. This finding suggests that SNIP may be particularly sensitive not only for early detection, but also for monitoring treatment response in LOPD.

Diaphragmatic ultrasonographic evaluation has gained popularity as a valuable tool in the assessment of diaphragm in injuries of the phrenic nerve and neuromuscular diseases [19, 28], particularly through measurement of the TF. Changes in diaphragmatic thickness in inspiration and expiration (this is TF), correlates well with inspiratory strength (related with changes in FVC and MIP) [29]. Moreover, it has been described that in slowly progressive myopathies, such as LOPD, ultrasound can be a sensitive test to detect diaphragmatic involvement and nocturnal hypercapnia [30], but measuring the diaphragm excursion velocity and not the TF itself. Diaphragmatic excursion may be difficult to measure if the patient already has established diaphragmatic atrophy. In both situations, the muscle may be too elevated to be properly studied by diaphragmatic excursion. Because of this, some authors consider the TF evaluation, since it allows to visualize the muscle regarding its situation in the thorax. We demonstrated a significant and continuous annual reduction in TF (− 6.6%, *p* = 0.03), supporting its superior sensitivity over traditional spirometry indices.

We observed that MIP, SNIP and TF showed a statistically significant progressive decrease much more evident than FVC alone. This could suggest that there are far more sensitive tools to diagnose respiratory involvement in patients with LOPD than FVC and, since the evidence of diaphragmatic weakness secondary to LOPD is an indication for treatment, a more exhaustive and multidimensional respiratory evaluation should be recommended for all LOPD patients. This multimodal approach could also be valuable in individuals identified through newborn screening who are apparently pre-symptomatic, as it may help detect early respiratory involvement and guide the timing of therapeutic interventions.

Weakness typically begins in the trunk and lower limb muscles, followed by involvement of the diaphragm and other respiratory muscles [31]. Respiratory involvement can occur early and sometimes independently of limb muscle weakness. Notably, severe respiratory failure has been reported in ambulatory LOPD patients with preserved limb strength [22]. This has been reported in a series of 29 patients with LOPD with severe respiratory failure without affectation of limb muscles [32], and this is why respiratory function should be monitored independently from the degree of muscle weakness [33].

In fact, in this series a single patient with a respiratory debut was included. The patient developed a clear diaphragmatic weakness with orthopnea, MIP 21%, SNIP 28 cmH_2_O and TF 30% while still ambulant and with preserved FVC 79%. Nocturnal capnography later confirmed hypoventilation, and the patient was treated with NIV with excellent clinical and ventilatory response. We believe that this case highlights the importance of assessing LOPD patients with a multi-dimensional respiratory approach, including not only complete pulmonary function tests but also diaphragmatic ultrasound and sleep studies like nocturnal capnography, to make a proper and precocious detection of respiratory failure in this disease.

The study has certain limitations, primarily related to the extraordinary healthcare disruptions caused by the COVID-19 pandemic. The full five-year follow-up could not be completed for all patients due to the temporary closure of outpatient clinics, staff shortages, and restrictions on patient mobility. Additionally, although the original study design included in-hospital nocturnal capnography, this was not feasible during the pandemic due to limited bed availability and institutional recommendations to minimize exposure risks for vulnerable patients. In addition, a potential selection bias should be acknowledged, as at the time of diagnosis at least 33% of the included patients already exhibited a restrictive pattern. Furthermore, some patients-initiated treatment with avalglucosidase alfa during the final months of the study. Therefore, the long-term effects of this newer formulation could not be fully assessed and warrant further evaluation in this subgroup. Another potential limitation is that newer ERT formulations were not available in our country during most of the study period. Therefore, most patients initially received alglucosidase alfa as first-line therapy, although some patients were switched to alternative formulations during follow-up. The relatively small cohort size, which reflects the rarity of LOPD and the single-center design. Despite these constraints, the study offers several notable strengths. It represents one of the longest prospective follow-ups of respiratory function in LOPD to date, employing a comprehensive and multidimensional assessment strategy, using validated, reproducible functional and imaging measures. Furthermore, the use of GLMMix strengthened the statistical robustness of the longitudinal analysis, enabling individualized tracking of disease progression while accounting for baseline variability. These methodological features enhance the validity and clinical applicability of the findings.

## Conclusions

In this prospective cohort study of patients with LOPD, we observed a slow but progressive decline in respiratory function over time. Given the availability of disease-modifying therapy (ERT) and the established association between respiratory function and prognosis. While FVC remains a standard parameter, our results demonstrate that TF and SNIP are significantly more sensitive indicators of early respiratory involvement.

## Supplementary Information

Below is the link to the electronic supplementary material.

Supplementary Material 1

## Data Availability

The datasets generated and/or analyzed during the current study are available from the corresponding author on reasonable request.

## Declarations

### Artificial intelligence

Artificial intelligence tools were used in the preparation of this manuscript for grammar correction and language refinement. All content was subsequently reviewed and validated by the authors to ensure accuracy and scientific integrity.

## References

[1] Labella B, Cotti Piccinelli S, Risi B, Caria F, Damioli S, Bertella E, et al. A Comprehensive Update on Late-Onset Pompe Disease. Biomolecules. 2023;13:1279. 10.3390/biom13091279. 37759679 10.3390/biom13091279 PMC10526932

[2] Porcino M, Musumeci O, Usbergo C, Pugliese A, Arena IG, Rodolico C, et al. Management of presymptomatic juvenile patients with late-onset Pompe disease (LOPD). Neuromuscul Disord. 2025;47:105277. 10.1016/j.nmd.2025.105277. 39879733 10.1016/j.nmd.2025.105277

[3] Nabatame S, Taniike M, Sakai N, Kato-Nishimura K, Mohri I, Kagitani-Shimono K, et al. Sleep disordered breathing in childhood-onset acid maltase deficiency. Brain Dev. 2009;31:234–9. 10.1016/j.braindev.2008.03.007. 18495398 10.1016/j.braindev.2008.03.007

[4] DeRuisseau LR, Fuller DD, Qiu K, DeRuisseau KC, Donnelly WH, Mah C, et al. Neural deficits contribute to respiratory insufficiency in Pompe disease. Proc Natl Acad Sci U S A. 2009;106:9419–24. 10.1073/pnas.0902534106. 19474295 10.1073/pnas.0902534106 PMC2695054

[5] Gambetti P, DiMauro S, Baker L. Nervous system in Pompe’s disease. Ultrastructure and biochemistry. J Neuropathol Exp Neurol. 1971;30:412–30. 10.1097/00005072-197107000-00008. 5284681 10.1097/00005072-197107000-00008

[6] Mancall EL, Aponte GE, Berry RG, POMPE’S DISEASE (DIFFUSE GLYCOGENOSIS) WITH NEURONAL STORAGE. J Neuropathol Exp Neurol. 1965;24:85–96. 10.1097/00005072-196501000-00008. 14253576 10.1097/00005072-196501000-00008

[7] Teng Y-T, Su W-J, Hou J-W, Huang S-F. Infantile-onset glycogen storage disease type II (Pompe disease): report of a case with genetic diagnosis and pathological findings. Chang Gung Med J. 2004;27:379–84. 15366815

[8] Thurberg BL, Lynch Maloney C, Vaccaro C, Afonso K, Tsai AC-H, Bossen E, et al. Characterization of pre- and post-treatment pathology after enzyme replacement therapy for Pompe disease. Lab Invest. 2006;86:1208–20. 10.1038/labinvest.3700484. 17075580 10.1038/labinvest.3700484

[9] Prigent H, Orlikowski D, Laforêt P, Letilly N, Falaize L, Pellegrini N, et al. Supine volume drop and diaphragmatic function in adults with Pompe disease. Eur Respir J. 2012;39:1545–6. 10.1183/09031936.00169011. 22654013 10.1183/09031936.00169011

[10] Mah C, Pacak CA, Cresawn KO, Deruisseau LR, Germain S, Lewis MA, et al. Physiological correction of Pompe disease by systemic delivery of adeno-associated virus serotype 1 vectors. Mol Ther. 2007;15:501–7. 10.1038/sj.mt.6300100. 17245350 10.1038/sj.mt.6300100

[11] Mah CS, Falk DJ, Germain SA, Kelley JS, Lewis MA, Cloutier DA, et al. Gel-mediated delivery of AAV1 vectors corrects ventilatory function in Pompe mice with established disease. Mol Ther. 2010;18:502–10. 10.1038/mt.2009.305. 20104213 10.1038/mt.2009.305 PMC2839425

[12] Forsha D, Li JS, Smith PB, van der Ploeg AT, Kishnani P, Pasquali SK, et al. Cardiovascular abnormalities in late-onset Pompe disease and response to enzyme replacement therapy. Genet Med. 2011;13:625–31. 10.1097/GIM.0b013e3182142966. 21543987 10.1097/GIM.0b013e3182142966 PMC3138812

[13] Merk T, Wibmer T, Schumann C, Krüger S. Glycogen storage disease type II (Pompe disease)--influence of enzyme replacement therapy in adults. Eur J Neurol. 2009;16:274–7. 10.1111/j.1468-1331.2008.02377.x. 19138339 10.1111/j.1468-1331.2008.02377.x

[14] van der Meijden JC, Güngör D, Kruijshaar ME, Muir ADJ, Broekgaarden HA, van der Ploeg AT. Ten years of the international Pompe survey: patient reported outcomes as a reliable tool for studying treated and untreated children and adults with non-classic Pompe disease. J Inherit Metab Dis. 2015;38:495–503. 10.1007/s10545-014-9751-2. 25112389 10.1007/s10545-014-9751-2

[15] Güngör D, Kruijshaar ME, Plug I, D’Agostino RB, Hagemans MLC, van Doorn PA, et al. Impact of enzyme replacement therapy on survival in adults with Pompe disease: results from a prospective international observational study. Orphanet J Rare Dis. 2013;8:49. 10.1186/1750-1172-8-49. 23531252 10.1186/1750-1172-8-49 PMC3623847

[16] Hagemans MLC, Hop WJC, Van Doorn PA, Reuser AJJ, Van der Ploeg AT. Course of disability and respiratory function in untreated late-onset Pompe disease. Neurology. 2006;66:581–3. 10.1212/01.wnl.0000198776.53007.2c. 16505317 10.1212/01.wnl.0000198776.53007.2c

[17] El Haddad L, Khan M, Soufny R, Mummy D, Driehuys B, Mansour W, et al. Monitoring and Management of Respiratory Function in Pompe Disease: Current Perspectives. Ther Clin Risk Manag. 2023;19:713–29. 10.2147/TCRM.S362871. 37680303 10.2147/TCRM.S362871 PMC10480292

[18] Katzin LW, Amato AA. Pompe disease: a review of the current diagnosis and treatment recommendations in the era of enzyme replacement therapy. J Clin Neuromuscul Dis. 2008;9:421–31. 10.1097/CND.0b013e318176dbe4. 18525427 10.1097/CND.0b013e318176dbe4

[19] Sayas Catalán J, Hernández-Voth A, Villena Garrido MV. Diaphragmatic Ultrasound: An Innovative Tool Has Become Routine. Arch Bronconeumol (Engl Ed). 2020;56:201–3. 10.1016/j.arbres.2019.06.020. 31383496 10.1016/j.arbres.2019.06.020

[20] Corbett M, Umemneku-Chikere C, Nevitt S, Deng NJ, Walton M, Fulbright H, et al. Enzyme replacement therapy for the treatment of late onset Pompe disease: A systematic review and network meta-analysis. Orphanet J Rare Dis. 2025;20:451. 10.1186/s13023-025-03981-0. 40842017 10.1186/s13023-025-03981-0 PMC12372379

[21] Hirschhorn R, Huie ML. Frequency of mutations for glycogen storage disease type II in different populations: the delta525T and deltaexon 18 mutations are not generally common in white populations. J Med Genet. 1999;36:85–6. 9950376 PMC1762954

[22] Mellies U, Stehling F, Dohna-Schwake C, Ragette R, Teschler H, Voit T. Respiratory failure in Pompe disease: treatment with noninvasive ventilation. Neurology. 2005;64:1465–7. 10.1212/01.WNL.0000158682.85052.C0. 15851748 10.1212/01.WNL.0000158682.85052.C0

[23] Laforêt P, Nicolino M, Eymard PB, Puech JP, Caillaud C, Poenaru L, et al. Juvenile and adult-onset acid maltase deficiency in France: genotype-phenotype correlation. Neurology. 2000;55:1122–8. 10.1212/wnl.55.8.1122. 11071489 10.1212/wnl.55.8.1122

[24] van der Beek Na, van Capelle ME, van der Velden-van Etten CI, Hop KI, van den Berg WCJ, Reuser B. Rate of progression and predictive factors for pulmonary outcome in children and adults with Pompe disease. Mol Genet Metab. 2011;104:129–36. 10.1016/j.ymgme.2011.06.012. 21763167 10.1016/j.ymgme.2011.06.012

[25] Morgan RK, McNally S, Alexander M, Conroy R, Hardiman O, Costello RW. Use of Sniff nasal-inspiratory force to predict survival in amyotrophic lateral sclerosis. Am J Respir Crit Care Med. 2005;171:269–74. 10.1164/rccm.200403-314OC. 15516537 10.1164/rccm.200403-314OC

[26] Murray D, Rooney J, Meldrum D, Al-Chalabi A, Bunte TM, Chiwera T, et al. Respiratory measurements, respiratory symptoms, and quality of life in ALS: results from the REVEALS study. Amyotroph Lateral Scler Frontotemporal Degener. 2025;26:467–77. 10.1080/21678421.2025.2471421. 40047382 10.1080/21678421.2025.2471421

[27] Yüksel Kalyoncu M, Gokdemir Y, Yilmaz Yegit C, Yanaz M, Gulieva A, Selcuk M, et al. Can Sniff Nasal Inspiratory Pressure be a guide in detecting of sleep-disordered breathing in children with Duchenne Muscular Dystrophy? Sleep Med. 2024;124:662–8. 10.1016/j.sleep.2024.10.004. 39531786 10.1016/j.sleep.2024.10.004

[28] Matamis D, Soilemezi E, Tsagourias M, Akoumianaki E, Dimassi S, Boroli F, et al. Sonographic evaluation of the diaphragm in critically ill patients. Technique and clinical applications. Intensive Care Med. 2013;39:801–10. 10.1007/s00134-013-2823-1. 23344830 10.1007/s00134-013-2823-1

[29] Summerhill EM, El-Sameed YA, Glidden TJ, McCool FD. Monitoring recovery from diaphragm paralysis with ultrasound. Chest. 2008;133:737–43. 10.1378/chest.07-2200. 18198248 10.1378/chest.07-2200

[30] Spiesshoefer J, Lutter R, Kabitz H-J, Henke C, Herkenrath S, Randerath W, et al. Respiratory Muscle Function Tests and Diaphragm Ultrasound Predict Nocturnal Hypoventilation in Slowly Progressive Myopathies. Front Neurol. 2021;12:731865. 10.3389/fneur.2021.731865. 34721265 10.3389/fneur.2021.731865 PMC8551547

[31] Winkel LPF, Hagemans MLC, van Doorn PA, Loonen MCB, Hop WJC, Reuser AJJ, et al. The natural course of non-classic Pompe’s disease; a review of 225 published cases. J Neurol. 2005;252:875–84. 10.1007/s00415-005-0922-9. 16133732 10.1007/s00415-005-0922-9

[32] Pellegrini N, Laforet P, Orlikowski D, Pellegrini M, Caillaud C, Eymard B, et al. Respiratory insufficiency and limb muscle weakness in adults with Pompe’s disease. Eur Respir J. 2005;26:1024–31. 10.1183/09031936.05.00020005. 16319331 10.1183/09031936.05.00020005

[33] Mellies U, Lofaso F. Pompe disease: a neuromuscular disease with respiratory muscle involvement. Respir Med. 2009;103:477–84. 10.1016/j.rmed.2008.12.009. 19131232 10.1016/j.rmed.2008.12.009

