# Supplementary material for: Multidimensional assessment of diaphragmatic dysfunction in late-onset Pompe disease: a prospective cohort study
Source: Orphanet J Rare Dis. 2026 Apr 7;21:204. doi: 10.1186/s13023-026-04343-0 (PMC13188754; doi:10.1186/s13023-026-04343-0)
Supplement: Supplementary file 1 — Supplementary Material 1 [file 13023_2026_4343_MOESM1_ESM.docx]

**Supplementary Material 1.** Spaghetti plots showing individual patient trajectories of forced vital capacity (FVC, % predicted), supine FVC decline (%), and diaphragmatic thickening fraction (TF, %) across follow-up visits labelled from baseline (months).


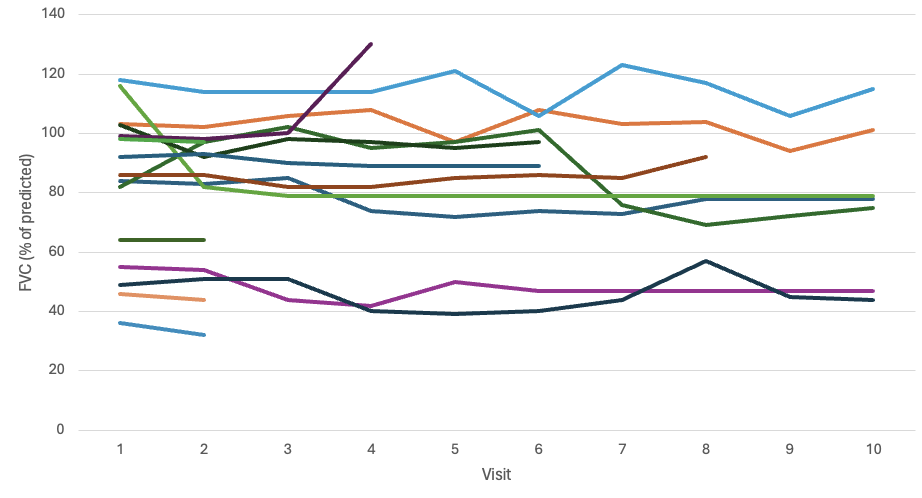


Basal +6m +12m +18m +24m +30m +36m +42m +48m +54m

Time (months)


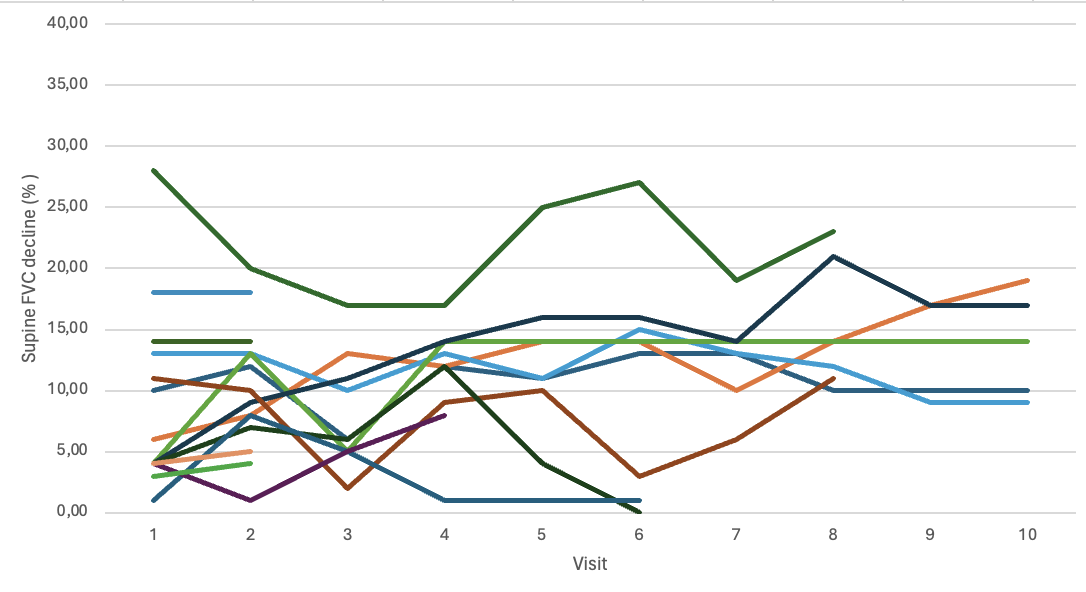


Basal +6m +12m +18m +24m +30m +36m +42m +48m +54m

Time (months)


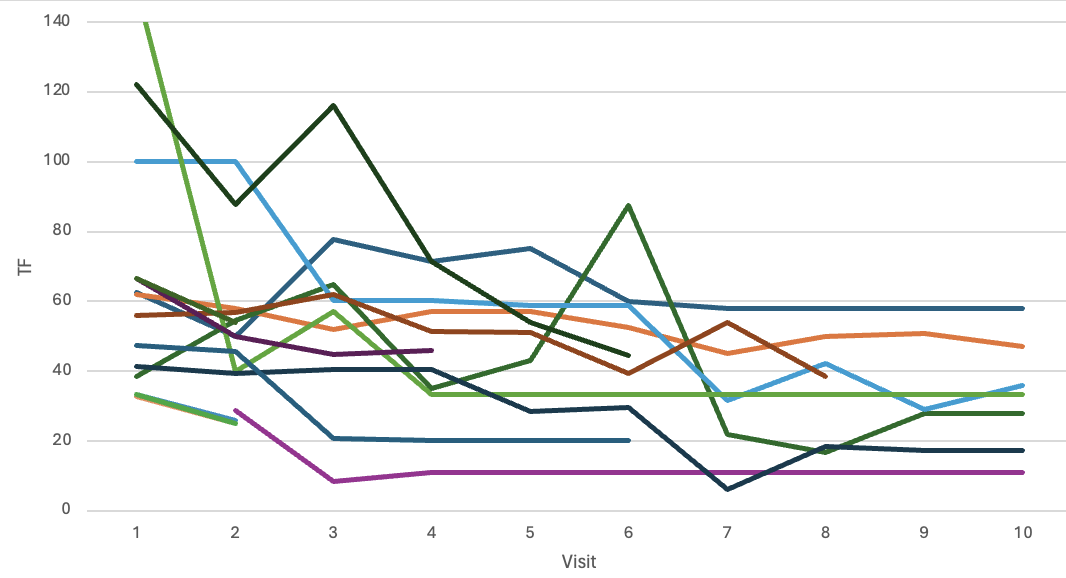


Basal +6m +12m +18m +24m +30m +36m +42m +48m +54m

Time (months)
